# Low-cost fabrication methods of ZnO nanorods and their physical and photoelectrochemical properties for optoelectronic applications

**DOI:** 10.1038/s41598-024-73352-5

**Published:** 2024-10-11

**Authors:** Mabrouk Bakry, Walid Ismail, Mahmoud Abdelfatah, Abdelhamid El-Shaer

**Affiliations:** 1 Physics Department, Faculty of Science, Kafrelsheikh University, Kafrelsheikh, 33516 Egypt; 2 NanoScience and Technology Program, Faculty of Science, Kafrelsheikh University, Kafrelsheikh, 33516 Egypt

**Keywords:** Low-cost, Hydrothermal, Chemical bath deposition, Electrochemical deposition, ZnO nanorods, Physical and photoelectrochemical properties, Optoelectronic applications, Engineering, Materials science, Optics and photonics, Physics

## Abstract

Zinc Oxide (ZnO) nanorods have great potential in several applications including gas sensors, light-emitting diodes, and solar cells because of their unique properties. Here, three low cost and ecofriendly techniques were used to produce ZnO nanorods on FTO substrates: hydrothermal, chemical bath deposition (CBD), and electrochemical deposition (ECD). This study explores the impact of such methods on the optical, structural, electrical, morphological, and photoelectrochemical properties of nanorods using various measurements. XRD analysis confirmed the hexagonal wurtzite structure of ZnO nanorods in all three methods, with hydrothermal showing a preferred orientation (002) and CBD and ECD samples showing multiple growth directions, with average particle sizes of 31 nm, 34 nm, and 33 nm, respectively. Raman spectra revealed hexagonal Wurtzite structure of ZnO, with hydrothermal method exhibiting higher E_2_ (high) peak at 438 cm^−1^ than CBD and ECD methods. SEM results revealed hexagonal ZnO nanorods became more regular and thicker for the hydrothermal method, while CBD and ECD led to less uniform with voids. UV-vis spectra showed absorption lines between 390 nm and 360 nm. Optical bandgap energies were calculated as 3.32 eV, 3.22 eV, and 3.23 eV for hydrothermal, CBD, and ECD samples, respectively. PL spectra revealed UV emission band with a small intensity peak around 389 nm and visible emission peaks at 580 nm. Temperature dependent PL measurements for ZnO nanorods indicated that the intensities ratio between bound exciton and free exciton decreases with temperature increases for the three methods. Photocurrent measurements revealed ZnO nanorod films as n-type semiconductors, with photocurrent values of 2.25 µA, 0.28 µA, and 0.3 µA for hydrothermal, CBD, and ECD samples, and photosensitivity values of 8.01, 2.79, and 3.56 respectively. Our results suggest that the hydrothermal method is the most effective approach for fabricating high-quality ZnO nanorods for optoelectronic applications.

## Introduction

ZnO nanorods have garnered a lot of interest lately due to their unique characteristics and wide range in many applications^1^. In nanotechnology, one-dimensional nanostructures are remarked for their remarkable electrical, optical, and mechanical properties making them ideal for various technological innovations^2^. ZnO nanorods’ huge surface area, wide band gap, strong exciton binding energy, and high aspect ratio make them very advantageous for usage in solar cells^3^, light-emitting diodes^4^, photodetectors^5^, photocatalysts^6^, and biosensors^7^. ZnO is a non-toxic, chemically stable, and accessible material abundant in the earth^8–10^. ZnO is an extrinsic semiconductor known as an n-type semiconductor with a bandgap of almost 3.37 eV and has high exciton-binding energy (60 meV)^10–13^. Recently, the preparation of ZnO nanostructures as nanowires, nanorods, nanotubes, and nanoflowers has gained growing attention^8,14–16^. Physical techniques like molecular beam epitaxy^17^, metal-organic chemical vapor deposition^18^, sputtering^19^, and pulsed laser deposition^20^ can be used to produce ZnO. However, such methods are expensive due to the requirement for high temperatures and low pressures, and equipment complexity. On the other hand, simplifying their manufacturing processes leads to high technical developments for industrial applications. So chemical methods such as hydrothermal deposition^21^, chemical bath deposition^22^, and electrochemical deposition^13^ are cost-effective, large-scale production potential and highly efficient for manufacturing nanoparticles for various applications.

Asmaa Al-Rasheedi et al. studied salt concentration on ZnO nanorods using hydrothermal methods. They found that higher salt concentrations increased rod diameter and crystallite size but reduced length, as confirmed by XRD analysis^21^. Min Wang and colleagues synthesized Cu-Ni co-doped ZnO nanorods on FTO substrates via a hydrothermal method. The co-doped nanorods were grown with added nickel and copper nitrates, enhanced photocatalytic degradation by generating electron-hole pairs under UV light, leading to increased free-carrier concentration and improved azo dye degradation^23^. (A) Kathalingam et al. used a unique chemical solution approach to synthesis ZnO nanostructures using different precursors and investigated the effects of bath concentration and temperature on ZnO nanostructures. By varying the HMT content, rod size and shape might be altered. Seamless long nanorods with a sheet-like shape were created using the zinc acetate and NaOH method. By manipulating the size and shape of the nanorods, the work was able to fabricate two terminal planar nanorod devices with distinct I-V nature^24^. L.V. Podrezova and colleagues used the hydrothermal method to grow ZnO nanorods on various conductive substrates, including metals like Cu, Ni, Pt, Ag, Au, and FTO. They prepared a ZnO seed layer using sol-gel spin coating and a growth solution containing zinc nitrate hexahydrate, hexamethylenetetramine, polyethylenimine, and ammonium hydroxide. The synthesis was carried out at 88 °C for 2 h. The resulting ZnO nanorods were vertically aligned, with the most ordered arrays observed on Au substrates^25^. Pooja (B) More et al. synthesized ZnO thin films on FTO substrates using a chemical bath deposition method. The films had a hexagonal wurtzite structure and rod structures with an average height of 630 nm and width of 340 nm. The optical band gap was 3.1 eV, and electrochemical tests confirmed n-type conductivity with a charge carrier density of 8.55 × 10^18^ cm^−3^^22^. Büşra Altun and colleagues studied the effects of cobalt doping on ZnO thin film sensors, focusing on their structural, optical, and CO_2_ gas sensing properties. They doped ZnO with 1%, 3%, 5%, and 7% cadmium using chemical bath deposition. The 3% Cd-doped sensor had the highest CO_2_ response, fast response times, good selectivity, and high stability^26^. Sanghyeon Moon and colleagues created ZnO nanorods on FTO glasses and ZnO nanoparticle-coated FTO glasses using chemical bath deposition. SEM images showed nanorod widths of 500 nm on FTO and 100 nm on nanoparticle-coated FTO. Electrochemical impedance spectroscopy (EIS) revealed lower charge transfer resistance and faster reaction kinetics for ZnO nanorods on FTO glasses^27^. Dubey and Saravanam studied the impact of negative potentials (-0.5 to -1.5 V) on ZnO nanostructures grown on FTO substrates for dye-sensitized solar cells. They used a zinc chloride and potassium chloride solution, three electrode configurations, and annealed samples at 400 °C for 1 h. UV-vis spectra showed increased absorption and redshift with higher potentials, while fluorescence spectra showed maximum emission at 650 nm at -1.5 V^28^. Sigamani Saravanan and colleagues created ZnO nanostructures on a thin film coated FTO substrate using spin coating and electrochemical techniques. They prepared a transparent Zn-doped TiO_2_ solution and aged it for 24 h before depositing it on the FTO substrate. The samples were heated in a muffle furnace and then electrochemically deposited using a three-electrode setup. The samples were calcined again and characterized using XRD peaks for anatase TiO_2_ and ZnO’s hexagonal wurtzite structure. Raman spectroscopy revealed ZnO phonon modes at 444 cm^−1^^29^. Daniel Solís-Cortés and his team studied the growth of ZnO nanorods on different transparent conductive oxide (TCO) substrates, including FTO, ITO, and IZO. They used linear sweep voltammetry in a three-electrode electrochemical cell, maintaining a 70 °C electrolyte solution and deposition at -0.700 V vs. SCE for 3600 s. The results showed that ZnO density was highest on IZO (7.83 × 10⁸ cm⁻²), with nanorod diameters ranging from 225 to 259 nm. XRD and HRTEM confirmed that IZO supports denser and better-aligned ZnO nanorods due to its optimal nucleation sites and improved vertical alignment^30^.

In this work, three chemical processes; hydrothermal deposition, chemical bath deposition, and electrochemical deposition will be utilized to produce ZnO nanorods on FTO substrates and explore the impact of such method on the optical, structural, electrical, morphological, and photoelectrochemical properties of nanorods using various measurements. X-ray diffraction (XRD), Raman spectroscopy, scanning electron microscopy (SEM), UV-vis spectroscopy, photocurrent measurements, and photoluminescence spectroscopy (PL) at room temperature and different temperatures.

## Experimental methods

### Synthesis of ZnO NRs

Prior to manufacturing ZnO nanorods using three different methods; hydrothermal, chemical bath deposition, and electrochemical deposition, fluorine-doped tin oxide (FTO) substrates underwent a standard cleaning procedure. This involved a 10-minute ultrasonic bath with acetone, isopropanol, and deionized water (Milli-Q, 18 MΩ.cm at 25 °C), respectively. Then, the substrates were dried for 4 h at 100 °C.

#### Grow of ZnO nanorods by hydrothermal method

The hydrothermal method was chosen for growing ZnO nanorods on FTO substrates due to its precise control over growth conditions, low substrate protection, uniform, aligned, and crystalline nanorods, and its environmental friendliness and cost-effectiveness for large-scale production.

ZnO nanorods were synthesized on a 2 cm × 2 cm FTO substrate by a hydrothermal process without seed layers^13,31^. The Zn^2+^ ions source was prepared as a 40 mL solution of 2,975 g of 0.25 M zinc nitrate hexahydrate [Zn(NO_3_)_2_·6H_2_O, Sigma-Aldrich, 99%] with a 40 mL solution of 4.71 g of 2.1 M potassium hydroxide [KOH, Sigma-Aldrich, 99.5%]. The solution was mixed together for 10 min. and then transferred to a Teflon-lined autoclave where FTO substrates were placed vertically. The autoclave was then maintained at 150 °C for 16 h. Afterward, the grown samples were rinsed several times with distilled water and allowed to air-dry for additional characterization^32^.

#### Grow of ZnO nanorods by chemical bath deposition (CBD) method

The chemical bath deposition (CBD) method was chosen for fabricating ZnO nanorods on FTO substrates due to its simplicity, cost-effectiveness, and large-scale production accessibility. CBD operates at low temperatures and requires minimal equipment, allowing precise growth parameter control and nanorod property customization.

ZnO nanorods were created utilizing the procedure previously outlined in Sect. 2.1, but instead of using an autoclave, chemical bath deposition was employed^33,34^. In such method, the solution was transferred to a flask within a water bath at 80 °C for 4 h where substrates were vertically positioned^35^.

#### Grow of ZnO nanorods by electrochemical deposition method

The electrochemical deposition method was chosen for fabricating ZnO nanorods on FTO substrates due to its precise control over growth, customizable nanorod properties, adjustable size, density, and orientation, scalability, cost-effectiveness, and compatibility with various substrates due to its low-temperature operation.

ZnO nanorods were formed by electrochemical deposition where a three-electrode cell system was utilized in the experiment with the FTO substrate serving as the working electrode, Ag/AgCl as the reference electrode, and platinum wire as the counter electrode. The electrochemical deposition was carried out in a solution containing 0.017 g of 0.5 mM zinc chloride [ZnCl_2_, Sigma-Aldrich, 98%] and 1.86 g of 0.1 M potassium chloride [KCL, Sigma-Aldrich, 99%] with an applied voltage of − 0.7 V vs. Ag/AgCl electrode for one hour at 60 °C while stirring at 250 rpm^3,36,37^.

Based on the following processes at static-potential (E = -0.7 V/SCE), metal oxide is formed by a chemical reaction between the native hydroxides (OH-) and the metal cations in the solution^38^:

$$\begin{aligned}{O}_{2}+4e+2{H}_{2}O&\to 4O{H}^{-} \\ {Zn}^{2+}+2O{H}^{-}&\to ZnO+{H}_{2}O\end{aligned}$$

To summarize, this is

$${Zn}^{2+}+\frac{1}{2}{O}_{2}+2e\to ZnO$$

### Characterization techniques

X-ray diffraction (XRD, Shimadzu 6000) with 0.154 nm wavelength CuKα radiation. The XRD measurements were performed at a scan speed of 8.0°/min, a scan step of 0.02°, 40 kV, and 30 mA. And Raman spectroscopy (WITec alpha300 R setup with two laser sources; 532 nm, Max. 30 mW and 785 nm, Max. 133 mW) were used to investigate the crystal structures of our samples. A UV-Vis spectrophotometer (JASSCO V-630) was used to analyze the optical absorption spectra of the samples. A scanning electron microscope (JSM-651OLV) was employed to examine the samples’ morphology. The PL spectrophotometer (He-Cd laser, 325 nm, Max. 200 mW, and a Synapse CCD camera that was built into the HORIBA iHR320 spectrometer was used to record the spectra) was utilized to obtain valuable data on the samples’ quality and purity. The photocurrent was measured using a homemade setup that included a 200 W tungsten lamp for lighting, an illumination switch, a three-electrode cell with the FTO substrate serving as the working electrode, Ag/AgCl as the reference electrode, and platinum wire as the counter electrode, and a controlling Bio-logic system. Photocurrent measurements were performed at 0 Volts vis Ag/AgCl electrode in 0.5 M Na_2_SO_4_ solution that acted as the supporting electrolyte.

## Results and discussions

### XRD analysis

**Fig. 1:**
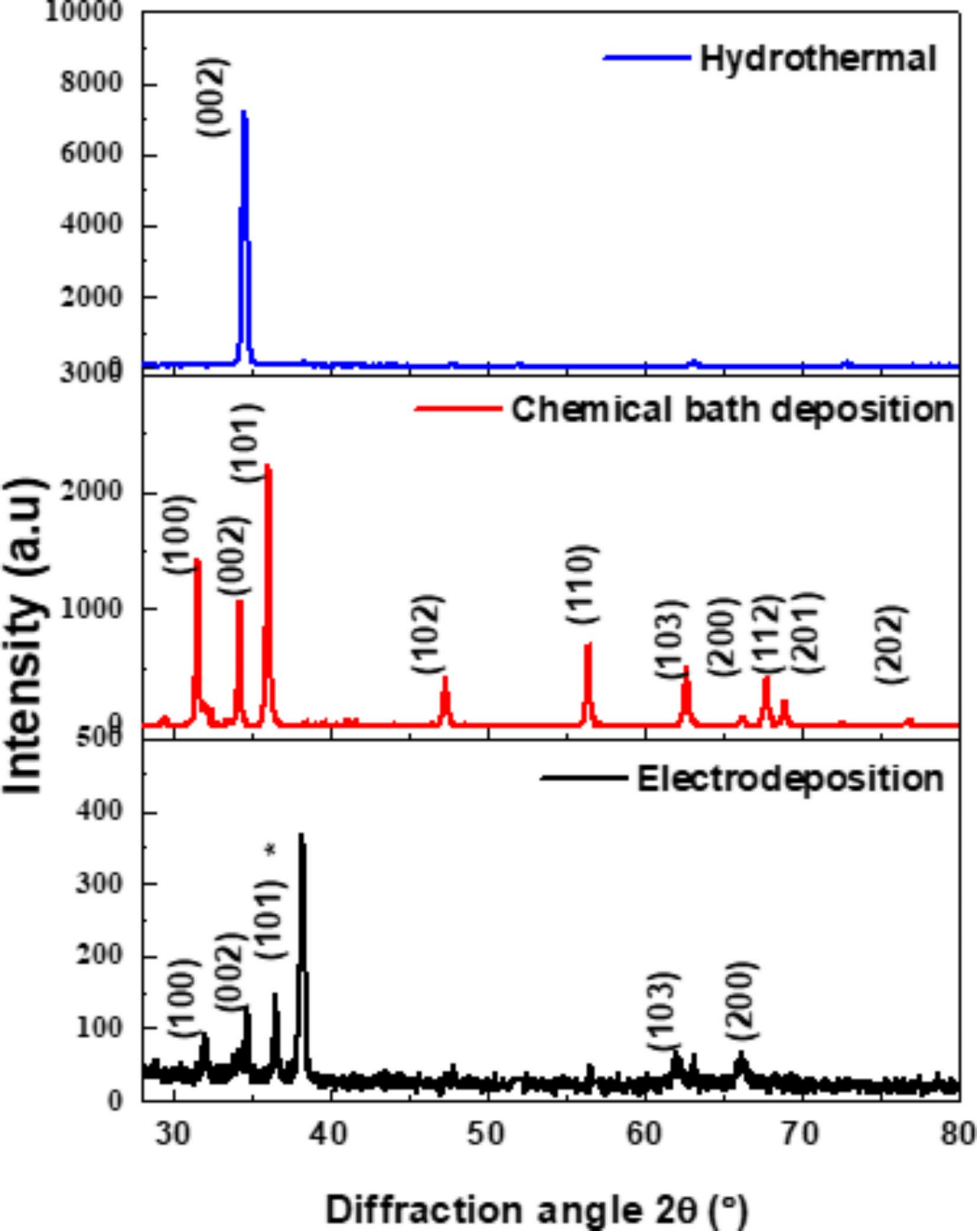
X-ray diffraction patterns of ZnO nanorods formed on FTO substrates using the following methods: (**a**) hydrothermal (**b**) chemical bath deposition (**c**) electrodeposition (* refers to FTO substrates).

XRD patterns of ZnO nanorods with the different methods (hydrothermal, CBD, and electrodeposition) are shown in Fig. 1. The diffraction patterns are very comparable to the hexagonal wurtzite structure of ZnO (standard JCPDS Card No 65-3411) with lattice constant (a = 0.325 nm, c = 0.521 nm)^39–42^. In the case of chemical bath deposition and electrodeposition methods, ZnO nanorods existed in (100), (002), (101), (103), and (200) direction planes meaning that ZnO nanorods are shaped on FTO substrates in various orientations. The FTO crystallographic planes are correlated with (JCPDS No.77–0452)^43^.

By the following equations, the lattice constants (a and c) of ZnO nanorods were obtained^44,45^:

$$\frac{1}{{d}^{2}}=\frac{4}{3}\left(\frac{{h}^{2}+hk+{k}^{2} }{{a}^{2}}\right)+\frac{{l}^{2}}{{c}^{2}}$$

when $k=l=0$, lattice parameter a was found from

$$a=\frac{\lambda }{\sqrt{3} \text{sin}\theta } \sqrt{{h}^{2}+hk+{k}^{2}}$$

when $h=k=0$, lattice parameter c was found from

$$c=\frac{\lambda }{2 \text{sin}\theta } l$$

where “d” is lattice spacing, $\lambda$ the wavelength of CuKα radiation, $\theta$ the Bragg diffraction angle in degree. It is found that (a = 0.3257 nm, c = 0.5226 nm) and (a = 0.333 nm, c = 0.516 nm) for chemical bath deposition and electrochemical deposition, respectively. For hydrothermal method, a high intensity preferred orientation (002) is noticed indicating the formation of ZnO nanorods perpendicular to FTO substrates in the c-axis direction with lattice constant c = 0.519 nm) due to high temperature promotes the creation of well-aligned nanorods by speeding up ZnO nucleation and development along this axis^46^. This can have an impact on the characteristics of ZnO by improving electron-hole pair radiative recombination, which can result in increased and more intense light emission, increased electron mobility, decreased recombination rates, and increased surface area.

The crystallite size has been calculated for ZnO nanorods by Scherer’s equation^47^:

$$D=\frac{K \lambda }{{\beta }_{hkl}\text{cos}\theta }$$

where λ is the wavelength of CuKα radiation, θ is the Bragg diffraction angle in radians, D is the crystallite size in nanometers, K is the shape of factor (0⋅9), and ${\beta }_{hkl}$ is the peak width at half-maximal intensity in radians. The average crystallite sizes D are determined for hydrothermal, CBD, and ECD methods are calculated in Table 1.

**Table 1:** The optical band gaps${E}_{g}$photocurrent (µA), average diameter (nm), and average crystallite size D (nm) of ZnO nanorods produced by hydrothermal, chemical bath deposition, and electrochemical deposition methods.

Method	${\varvec{E}}_{\varvec{g}}$ (eV)	Photocurrent (µA)	Average Diameter (nm)	D (nm)
Hydrothermal	3.32	2.26	180	31
Chemical bath	3.22	0.28	158	34
Electrochemical	3.23	0.30	146	33

The Zn-O bond length is obtained by equation^48^:

$$L= \sqrt{(\frac{{a}^{2}}{3}+{\left(\frac{1}{2}-u\right)}^{2}}*{c}^{2})$$

In the wurtzite structure, u is the positional parameter that signifies the amount that each atom is moved along the ‘c’ axis with the subsequent one. It is provided by:

$$u= \frac{{a}^{2}}{3*{c}^{2} }+0.25$$

The relationship between c/a and u indicates that when c/a declines, u increases in a way that keeps those four tetrahedral distances almost constant via a distortion of tetrahedral angles. The Zn-O bond length in the ZnO unit cell with surrounding atoms is 0.19767 nm^48^. The determined bond length of Zn-O is found to be 0.1983 nm and 0.2006 nm for chemical bath deposition and electrochemical deposition, respectively.

The determined bond length in the unit cell correlates with the Zn-O bond length. The results of XRD support the high purity of the ZnO nanorods synthesized and hence the strong crystalline nature because there are no impurity peaks.

### Raman spectroscopy analysis

Researchers can better understand transport characteristics and phonon interactions with free carriers which impact device performance by using Raman measurements which reveal material quality, phase, and purity^49^. Therefore, we apply Raman spectroscopy to study the structure of ZnO nanorods. The Wurtzite-type ZnO has two formula units in the primitive cell^50,51^, and belongs to the space group C^4^ (P63mc), and single-crystalline ZnO exhibits eight sets of optical phonon modes at $\Gamma$ point of the Brillouin zone, which are classified as^52,53^:

$$\Gamma_{opt}= 1{A}_{1}+2{B}_{I}+1{E}_{1}+2{E}_{2}.$$

A_1_ + E_1_ + 2E_2_ modes (Raman active), 2B_1_ modes (Raman silent), and A_1_ + E_1_ modes (Raman silent) (infrared active). Both the A_1_ and E_1_ modes are polar and split into transverse optical (TO) and longitudinal optical (LO) phonons.

**Fig. 2:**
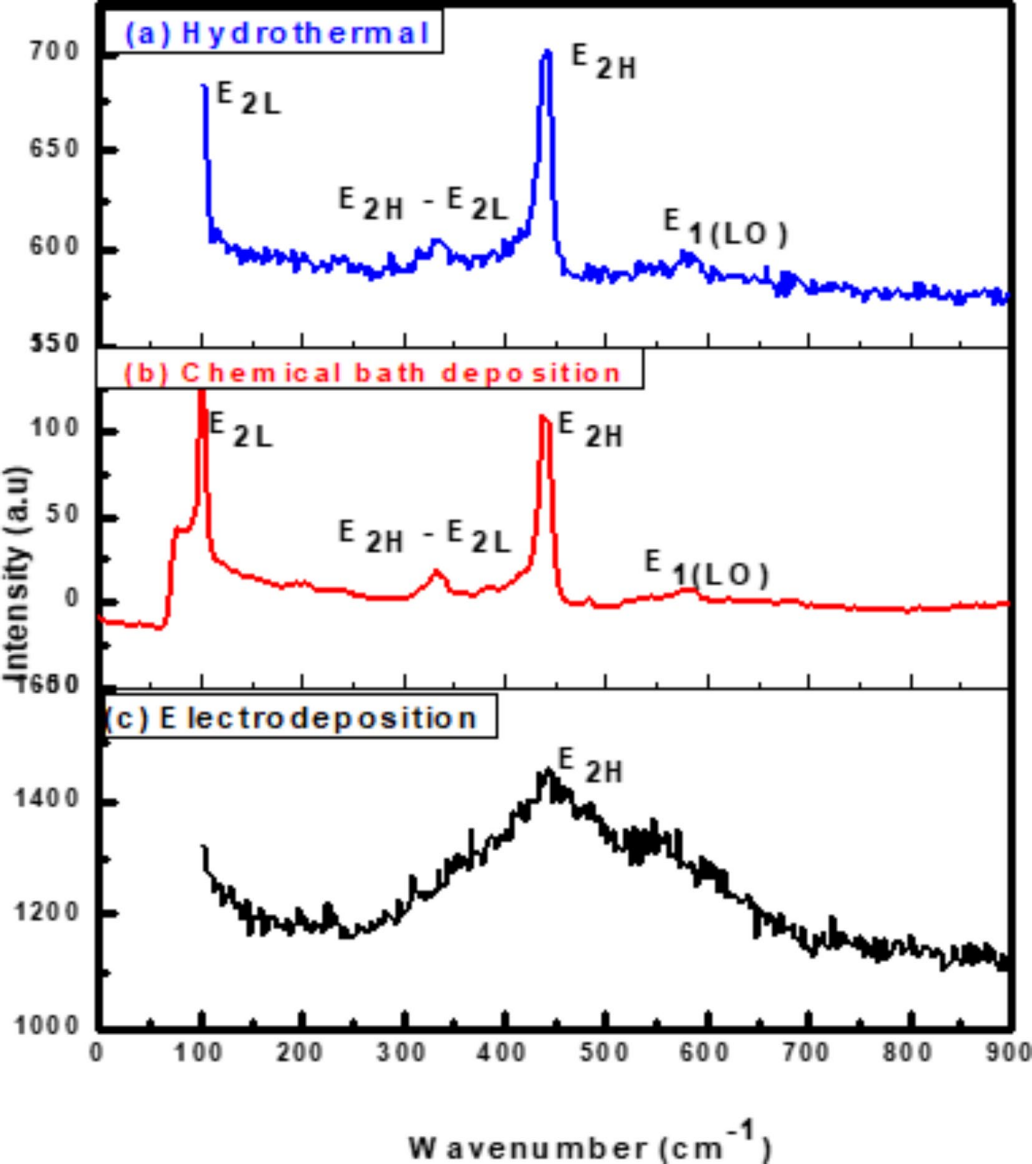
Raman scattering spectra of the ZnO NRs on FTO substrates prepared by (**a**) hydrothermal (**b**) chemical bath deposition (**c**) electrochemical deposition method.

Raman spectra of ZnO NRs using three different methods at room temperature are shown in Fig. 2. Raman shifts are similar to those previously published^53^ which are attributed to ZnO nanorods. The main peaks at 100 and 438 cm^−1^ are Raman active and commonly observed in the wurtzite structure ZnO^54^ which are attributed to the low- and high- E_2_ modes of nonpolar optical phonons, respectively. A second-order nonpolar E_2_ mode^55^ which is Raman active only, and has been attributed to the weaker peak at 333 cm^−1^. The E_2_(high) for the hydrothermal method is clearly visible at 438 cm^−1^, and it is much higher than E_2_ (high) for CBD and electrochemical deposition methods, also full width at half maximum of this peak for hydrothermal method is narrow in comparison with CBD and ECD methods showing good crystal quality of self - assembling radial structures, and resulting in a more ordered crystal structure^56^. It is not immediately clear that the peak at 411 cm^−1^ relates to the E_1(TO)_ mode. One of the distinctive features of the hexagonal wurtzite ZnO is the extremely powerful E_2_ (high) peak located at 438 cm^−1^. E_1(TO)_ on the left-hand side of E_2H_ is hidden by the asymmetrical and line-broadening properties. The E_1(LO)_ mode is responsible for the peak at 578 cm^−1^ which is induced by defects such as oxygen vacancy, zinc interstitial, or their complexes and free carriers^57^. As ZnO contains a significant number of intrinsic defects. In particular, many surface defects in rod ZnO nanostructures will lead to a distribution of surface energy levels constituting the surface energy band. The excitation of surface states can be induced by sub-band gap excitation^58^. As a result, the visible Raman emission could be related to surface states. These defects can influence the efficiency of light emission and detection, and can impact the charge separation and collection efficiency in solar cells. Based on the relative intensity of E_1(LO)_, we believe that electrochemical deposition has better crystal perfection than hydrothermal and CBD methods. In other words, the defect concentration in the hydrothermal method is somewhat larger than in CBD and electrochemical deposition methods.

### Analysis of Surface morphology

**Fig. 3:**
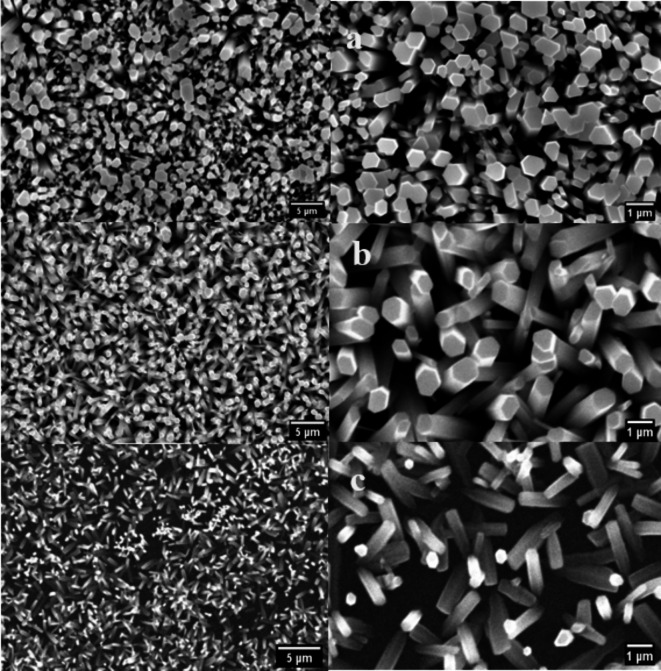
SEM images of ZnO nanorods grown on FTO substrates using (**a**) hydrothermal (**b**) chemical bath deposition (**c**) electrochemical deposition methods.

Figure 3 displays top-view SEM images of ZnO nanorods grown by hydrothermal, chemical bath deposition, and electrodeposition on FTO substrates. In the case of chemical bath deposition and electrodeposition methods, it is evident that ZnO was shaped in rod arrays with a hexagonal (wurtzite) structure and that the nanorods condensed to form nanorod shapes such as rod-like structures, not all the nanorods were perpendicular to the substrate where some voids appeared. This variation is due to the less controlled deposition environment and slower growth kinetics, which allow for more random growth directions and sizes [59, 60]. For hydrothermal method, the ZnO nanorods with hexagonal shapes become more regular and thicker where the voids vanished and nanorods cover the substrate’s surface with approximately one orientation along the c-axis on the FTO substrate [61]. This is because the hydrothermal conditions encourage the wurtzite structure’s development along its c-axis, producing well-ordered nanorods and a high aspect ratio. The average diameter of ZnO nanorods for three methods is calculated by using image J software as shown in table 1. The thickness and length of nanorods were calculated using the mass and density of samples before and after deposition and were found to be around 3, 2.5, and 1.5 μm. The XRD results have been confirmed by the SEM results where ZnO nanorods by chemical bath deposition and electrodeposition methods have a hexagonal structure with different orientation, while almost one c – axis orientation was observed for the hydrothermal method.

### UV-Vis studies

**Fig. 4:**
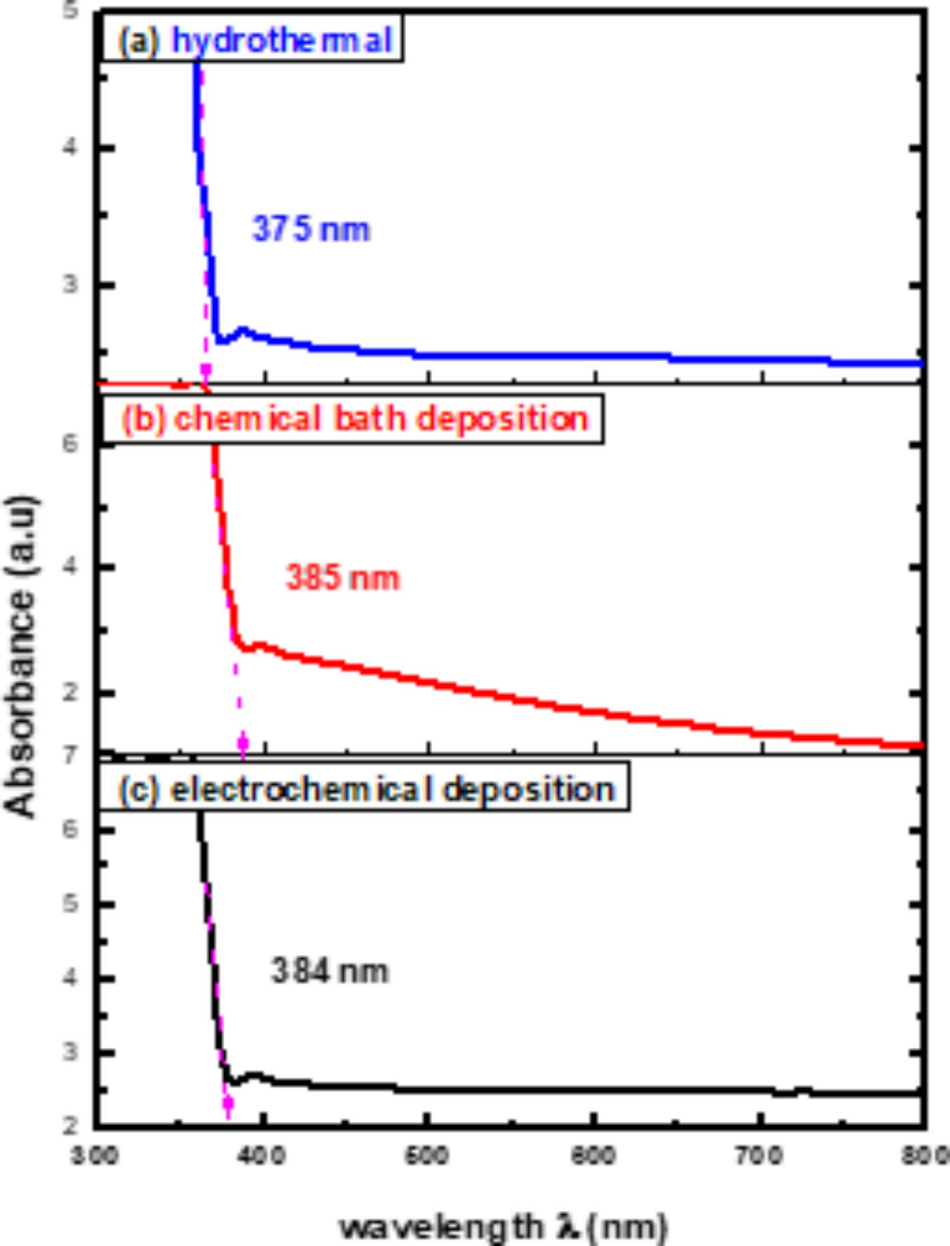
Absorption spectra of ZnO nanorods produced on FTO substrates by (**a**) hydrothermal (**b**) chemical bath deposition (**c**) electrochemical deposition methods.

V 630 spectrophotometer was used to investigate the optical characteristics of ZnO nanorods in a spectral range between 300 and 800 nm. Figure 4 displays UV- vis absorption spectra of ZnO nanorods where absorption peaks around 375 nm, 385 nm and 384 nm appeared for hydrothermal, chemical bath deposition, and electrochemical deposition, respectively. Such peaks are attributed to the movement of electrons between the valence band and the conduction band (O_2p_ – Zn_3d_), which corresponds to the band gap and absorption peaks of ZnO^62–64^.

**Fig. 5:**
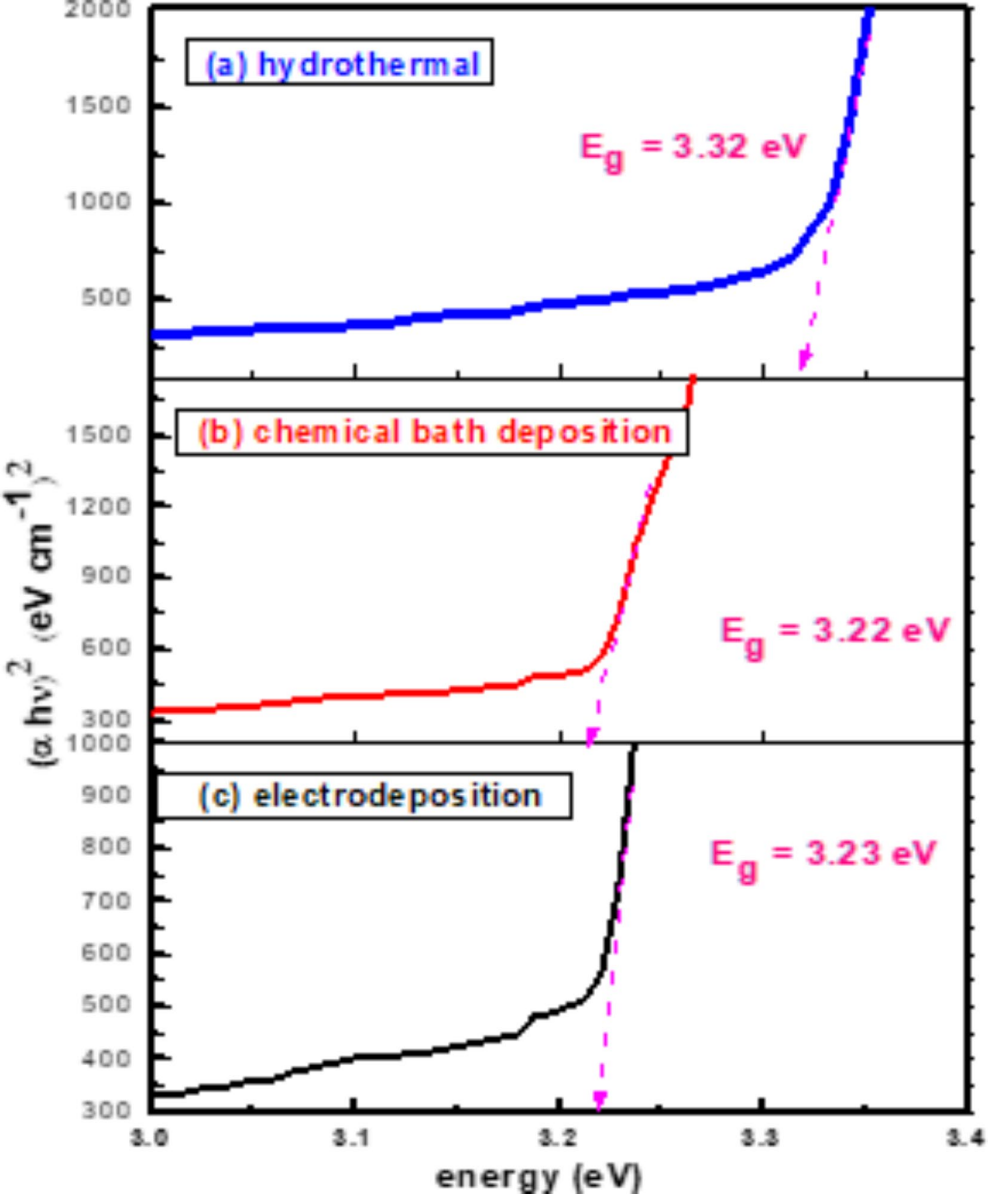
${\left(\varvec{\alpha }\varvec{h}\varvec{\nu }\right)}^{2}$vs. photon energy$\varvec{h}\varvec{\nu }$of ZnO nanorods produced on FTO substrates by (**a**) hydrothermal (**b**) chemical bath deposition (**c**) electrochemical deposition methods.

For direct band gap materials, the absorption coefficient could be calculated using The Tauc equation, ${\left(\alpha h\nu \right)}^{2}=K(h\nu - {E}_{g})$ at the absorbance edge. ${E}_{g}$ was estimated by extending the linear part of the dependency ${\left(\alpha h\nu \right)}^{2}$ on $h\nu$ to the energy axis^65–67^. The calculated optical band gaps of ZnO nanorods are found in Table 1 for the hydrothermal method, chemical bath deposition method, and electrochemical deposition method, respectively as shown in Fig. 5.

### Photoluminescence (PL) analysis

**Fig. 6:**
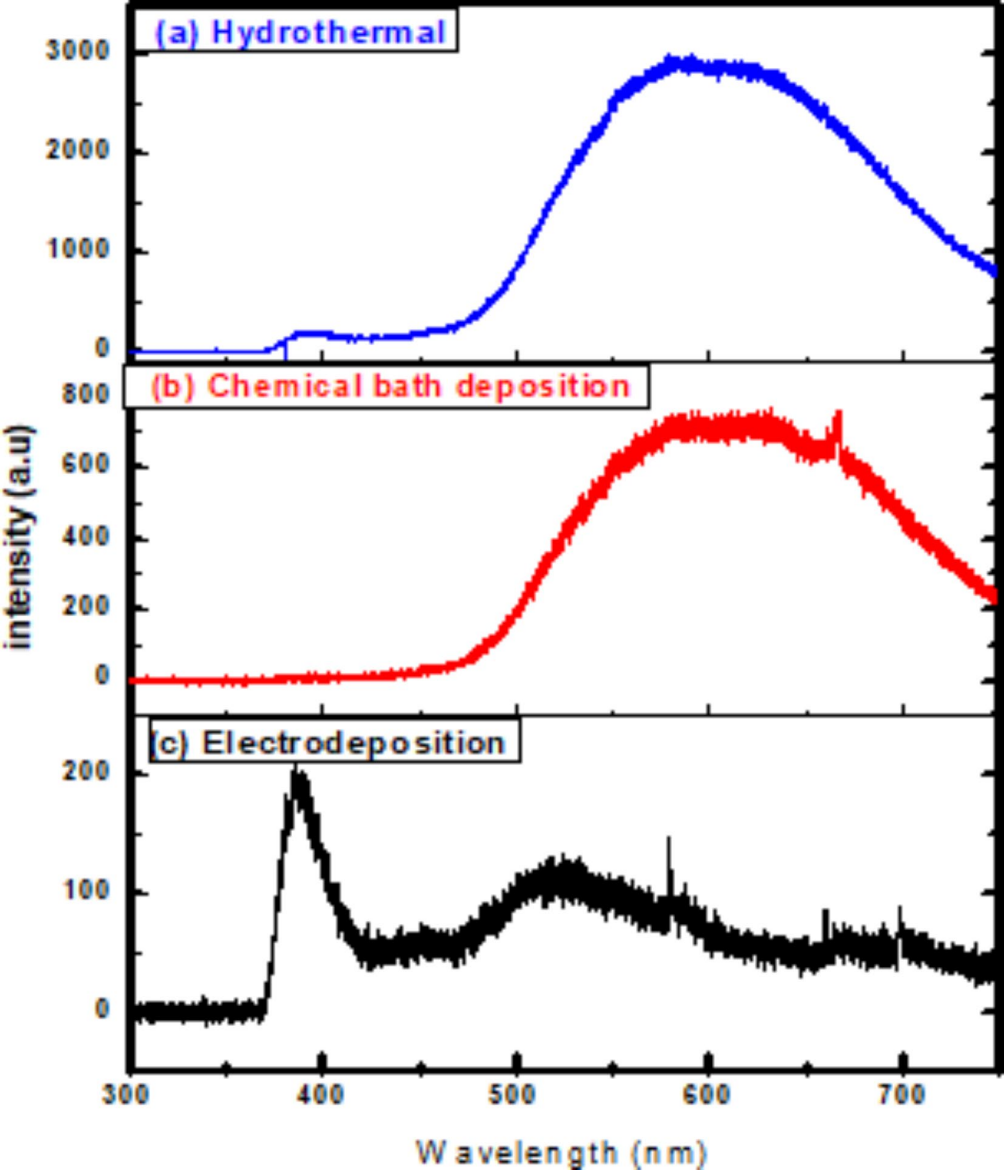
Room temperature PL of ZnO nanorods produced on FTO substrates by (**a**) hydrothermal (**b**) chemical bath deposition (**c**) electrochemical deposition methods.

PL is one of the most powerful semiconductor techniques for detecting the presence of defects where PL of ZnO nanorods was recorded at ambient temperature with excitation wavelength of 325 nm as shown in Fig. 6. Two emissions peaks are found in the spectrum of PL; UV and visible light ones with different relative strength of peaks and their location for different methods. UV emissions is referred to as Near Band Edge Emission of ZnO with a small peak at about 389 nm that could be resulted from the annihilation of the excitons as ZnO exhibits a high exciton binding energy at ambient temperature (60 meV)^68–70^. In the meantime, a broad emission peak in the visible range appeared due to the presence of deep level defects such as vacancies of single ionized oxygen and interstitial zinc or their complexes^71,72^. The large deep level emission and the weak UV band demonstrated the great potential of optoelectronic system applications for ZnO nanorods.

**Fig. 7:**
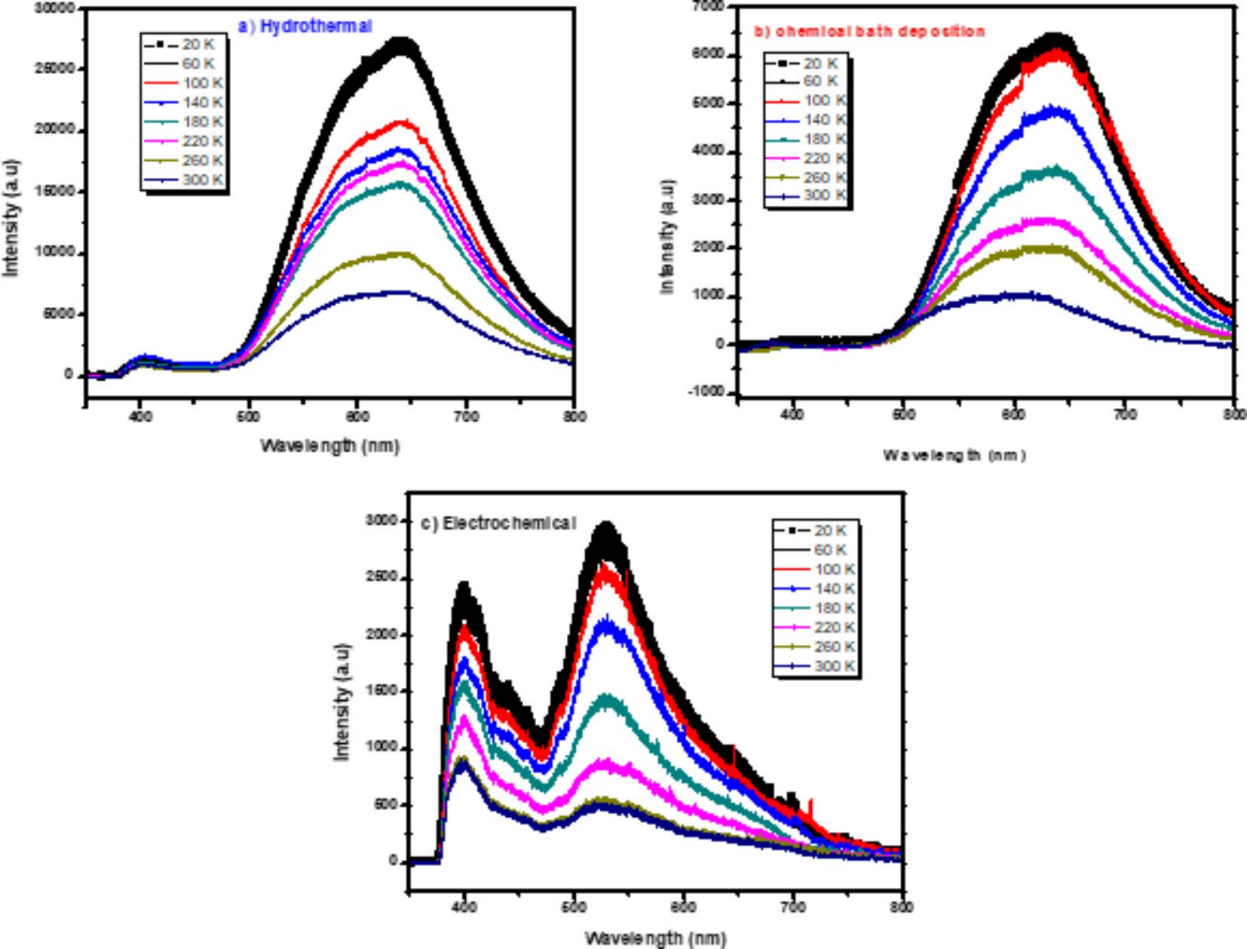
Temperature dependence PL of ZnO nanorods on FTO substrates by (**a**) hydrothermal (**b**) chemical bath deposition (**c**) electrochemical deposition methods.

In order to learn more about the nature of point defects in ZnO nanorods, temperature-dependent PL spectra were studied in detail as shown in Fig. 7 where the measurements were performed between 20 and 300 K. It is evident that the intensity ratio of bound to free exciton falls with increasing temperature. This is because the bound exciton transition process outweighs when the thermal energy is less than the binding energy and the free exciton transition process supersedes when the thermal energy is greater. We can also see how defective emissions behave differently as a function of temperature. With increasing temperature, the green, red, and yellow emission peaks shift, and the intensity of the defect emission decreases in ZnO NRs by three methods. In the case of a high defect concentration, a broad and featureless emission is predicted due to the formation of an energy band rather than discrete defect levels^73^.

### Photocurrent measurements

Photocurrent measurements were measured with a three-electrode electrochemical system with the FTO substrate serving as the working electrode, Ag/AgCl as the reference electrode, and platinum wire as the counter electrode. Where a broad-spectrum 200 W tungsten lamp illuminated the working electrode, manually switched on and off at predetermined intervals of time (30 s). The measurements were performed in 0.01 M sodium sulfate (Na_2_SO_4_). Bio-Logic Sb-50 potentiostat regulates both the applied voltage and scan rate at a bias potential of 0 V vs. Ag/AgCl where an electrochemical cell (PEC) was performed both in the dark and under light^73^.

**Fig. 8:**
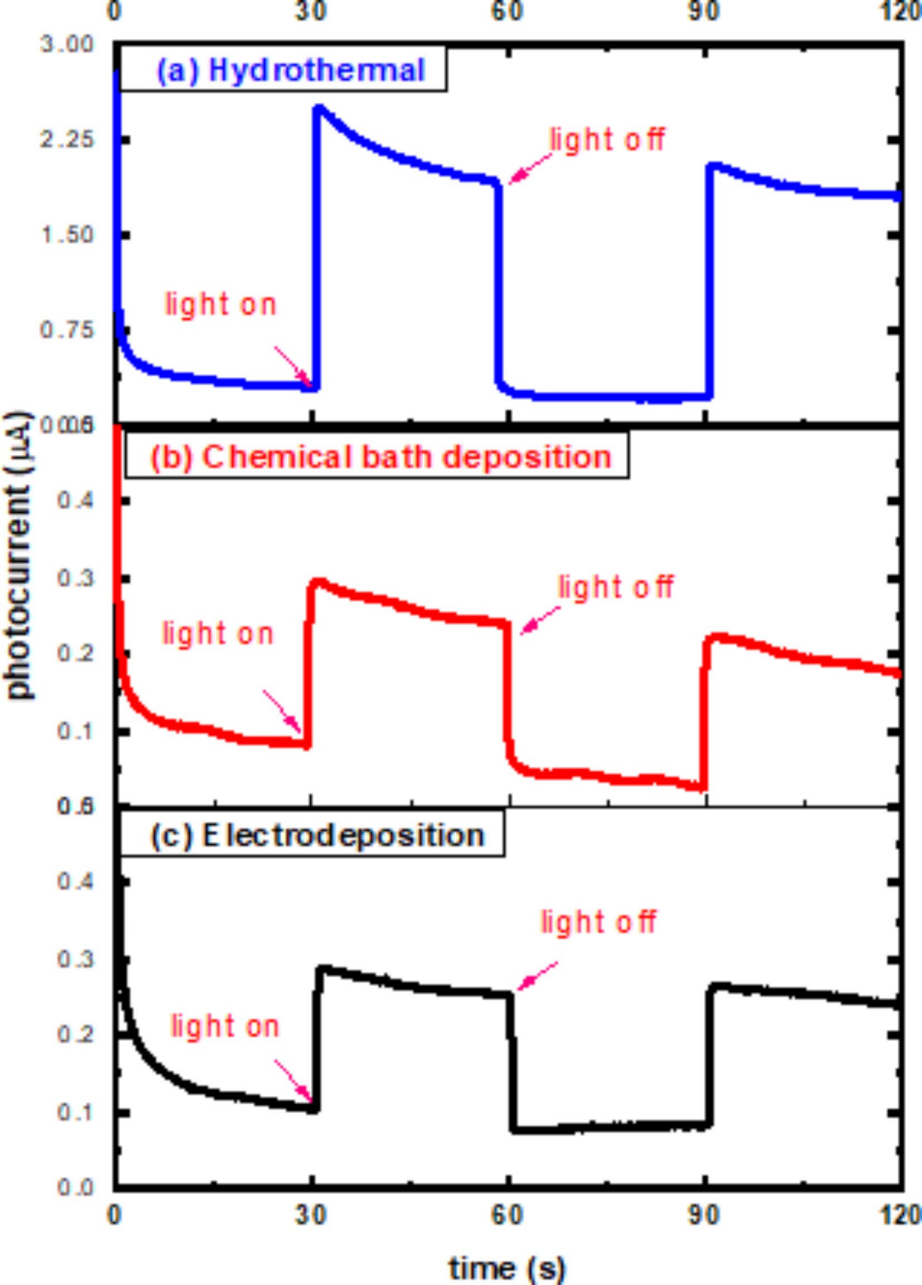
Photoconductivity rise and decay time spectra of ZnO nanorods produced on FTO substrates by (**a**) hydrothermal (**b**) chemical bath deposition (**c**) electrochemical deposition methods: photoconductive response due to visible light excitation for ZnO nanorods.

The rise and decline of photocurrent in air under visible light is shown in Fig. 8 for all samples with a constant photo flux and zero bias voltage. The cell was maintained dark at first until the dark current was stable. In comparison, the current density in the dark is near to zero. However, in the presence of light, the curve exhibits an almost instantaneous and constant reaction, with a significant increase in current. The material is still being exposed to light as it scatters inside the nanorods. When the photogenerated current is positive, n-type conductivity is present^74^. This could be attributed to the formation of additional negative charges on the semiconductor’s surface when the samples were exposed to light. This light activation likely generated electrons, which then moved from the electrolyte to the grown film, leading to the emergence of upward photocurrents^75,76^. Even during illumination, the photocurrent began to gradually decline after reaching a high. The delayed process of photoinduced chemisorption of oxygen molecules on the surface of ZnO nanostructures could explain this strange phenomenon^77^. When the irradiation is turned off, the photocurrent quickly drops to nearly zero, and it returns to normal as soon as the light is switched back on. This indicates that the current is entirely driven by the photoelectrode’s response to visible light and that the charge transport is extremely rapid^78–80^.

In photoconductivity measurements, the photosensitivity (η) is an important parameter which is defined as the maximal photocurrent (I_pc_) divided by the dark current (I_dc_). The photosensitivity illustrates the dependence of the defect state density on temperature, composition, and light intensity in the gap. The computed values of η for each sample at room temperature are listed in Table 2.

**Table 2:** The rise and decay time constants ${\tau }_{r}$ (s), ${\tau }_{d}$ (s), trap ionization energies (trap depth, eV) of different traps corresponding to different exponentials and photosensitivity η for three methods.

Method	${\varvec{\tau }}_{\varvec{r}}$ (s)	${\varvec{\tau }}_{\varvec{d}}$ (s)	Η	Trap depth (eV)
Hydrothermal	0.2	0.239	8.01	0.496
Chemical bath	0.346	0.839	2.79	0.508
Electrochemical	0.243	1.29	3.56	0.522

The value of η is determined by the excess carrier lifetime which is determined by the density of localized states in each material. Because defect states operate as recombination centers in the presence of light, the larger the density of defect states, the shorter the lifetime. As a result, the photosensitivity changes could be related to a change in defect state density with composition^81^.

The trap depths may be determined by separating the decaying part of the current rise and decay time response curves into the potential number of exponentials. The exponentials are calculated using the Eqs^82,83^:

$$I= {I}_{0}\text{exp}(-pt)$$

where ${I}_{0}$ represents the current when the light is turned off, I represents the photocurrent at any given time, and p is the probability of an electron escaping from the trap each second, which has a different value for each exponential section. The chance of an electron escaping from a trap can also be calculated using the following equation:

$$I= S\text{exp}(-E/KT)$$

where K is the Boltzmann constant (1.381 $\times$10^−23^ J/K), T is the absolute temperature, and S is the frequency factor that gives the rate at which quanta from crystal vibrations (phonons) try to eject electrons from traps multiplied by the probability of transition from trap to the conduction band and it has a value of 10^9^ at room temperature^84–86^.

Using the preceding equations, the trap depths (E) corresponding to various exponentials are determined and represented by:

$$E=KT\left[\text{ln}S-\text{ln}\frac{\text{ln}\left(\raisebox{1ex}{${I}_{0}$}\!\left/ \!\raisebox{-1ex}{$I$}\right.\right)}{t}\right]$$

The trap depth is calculated from the decay portion of the curve shown in Fig. 8 for all methods and its values are presented in Table 2.

## Conclusion

We have documented the development of vertically growing ZnO nanorods on FTO substrates using low cost and ecofriendly hydrothermal, chemical bath deposition and electrochemical processes. The structure, morphological, optical, and photoelectrochemical of the synthesized ZnO nanorods are investigated using XRD, Raman spectroscopy, SEM, UV-Vis, PL, and photocurrent measurements. XRD of ZnO films by hydrothermal method showed a high intensity preferred orientation (002), while chemical bath and electrochemical deposition methods shaped ZnO in various orientations. The Raman scattering spectra of ZnO NRs show that the hydrothermal method exhibited a higher E_2_ peak at 438 cm^-1^ where active in the wurtzite structure ZnO, indicating good crystal quality of self-assembling radial structures. SEM results showed that hexagonal ZnO nanorods became more regular and thicker with voids disappearing for hydrothermal methods. The UV-vis spectra showed absorption edges at 378 nm, 389 nm, and 366 nm, corresponding to electron transfer from the valence band to the conduction band of ZnO nanorods. PL results showed that ZnO nanorods emit UV and visible light peaks which produce exciton annihilation and deep defects. The temperature-dependent PL measurements indicated decreasing in intensity ratio between bound and free excitons. Photocurrent measurements showed n-type conductivity for all the fabricated samples where the hydrothermal method had more carriers due to increased current intensity. Our results indicate that the fabricated ZnO nanorods with the hydrothermal method is the preferred for optoelectronic applications.

## Data Availability

The datasets used and/or analyzed during the current study available from the corresponding author on reasonable request.
